# Effect of late gadolinium enhancement on left atrial impairment in myocarditis patients

**DOI:** 10.1007/s00330-023-10176-3

**Published:** 2023-09-02

**Authors:** Riccardo Cau, Giuseppe Muscogiuri, Francesco Pisu, Lorenzo Mannelli, Sandro Sironi, Jasjit S. Suri, Gianluca Pontone, Luca Saba

**Affiliations:** 1 Department of Radiology, Azienda Ospedaliero Universitaria (A.O.U.), di Cagliari – Polo di Monserrato s.s. 554 Monserrato, 09045 Cagliari, Italy; 2 School of Medicine and Surgery, University of Milano-Bicocca, Milan, Italy; 3 Department of Radiology, IRCCS Istituto Auxologico Italiano, San Luca Hospital, Milan, Italy; 4 Department of Radiology, ASST Papa Giovanni XXIII Hospital, Bergamo, Italy; 5 Stroke Monitoring and Diagnostic Division, AtheroPoint™, Roseville, CA USA; 6 Department of Perioperative Cardiology and Cardiovascular Imaging, Centro Cardiologico Monzino IRCCS, Milan, Italy; 7 Department of Biomedical, Surgical and Dental Sciences, University of Milan, Milan, Italy

**Keywords:** Myocarditis, Late gadolinium enhancement, Left atrium, Strain

## Abstract

**Objective:**

The aims of our study were to investigate the effect of the extent and location of late gadolinium enhancement (LGE) on the left atrium (LA) function in patients with acute myocarditis (AM) using cardiovascular magnetic resonance (CMR).

**Method:**

This retrospective study performed CMR scans in 113 consecutive patients (89 males, 24 females; mean age 45.8 ± 17.3 years) with AM that met the updated Lake Louise criteria. Reservoir, conduit, and booster LA functions were analyzed by CMR feature tracking using dedicated software. Besides LA strain measurements, myocardial scar location and extent were assigned and quantified by LGE imaging.

**Results:**

AM patients with septal LGE had impaired reservoir, conduit, and conduit strain rate function in comparison with AM patients with non-septal LGE (*p* = 0.001, for all). In fully adjusted multivariable linear regression, reservoir and conduit were significantly associated with left ventricle (LV) LGE location (*β* coefficient = 8.205, *p* = 0.007; *β* coefficient = 5.185, *p* = 0.026; respectively). In addition, LA parameters decreased according to the increase in the extent of LV fibrosis (LGE ≤ 10%; LGE 11–19%; LGE ≥ 20%). After adjustment in multivariable linear regression, the association with LV LGE extent was no longer statistically significant.

**Conclusion:**

In patients with acute myocarditis, LA function abnormalities are significantly associated with LV LGE location, but not with LGE extent. Septal LGE is paralleled by a deterioration of LA reservoir and conduit function.

**Clinical relevance statement:**

Left atrium dysfunction is associated with the presence of late gadolinium enhancement in the left ventricle septum and can be useful in the clinical prognostication of patients with acute myocarditis, allowing individually tailored treatment.

**Key Points:**

*• Myocardial fibrosis is related to atrial impairment.*

*• The location of myocardial fibrosis is the main determinant of atrial dysfunction in myocarditis patients.*

*• The quantification of atrial mechanisms may provide more in-depth insight into myocarditis pathophysiology.*

**Supplementary information:**

The online version contains supplementary material available at 10.1007/s00330-023-10176-3.

## Introduction

Acute myocarditis (AM) is an inflammatory disease of the myocardium with heterogenous variation in clinical presentation and outcomes [1–4]. While most patients recover completely, some developed potentially life-threatening complications resulting in dilated cardiomyopathy and heart failure [2, 5]. Cardiovascular magnetic resonance (CMR) is currently used as the reference standard non-invasive test in the diagnostic work-up of patients with suspected AM [2, 6–8]. In a recent meta-analysis, it was demonstrated that late gadolinium enhancement (LGE) presence, extent, and location on CMR are predictive of multiple adverse events, including all-cause mortality, cardiac mortality, and major adverse cardiovascular events [9]. However, the impact of LGE on myocardial function in patients with AM remains poorly understood.

Weber et al investigated the effect of myocardial injuries, defined by the presence of LGE on the change of left ventricle (LV) function reporting a regional myocardial dysfunction with a compensatory mechanism of the surrounding myocardium [10]. LV mechanism is impaired in patients with AM despite preserved ejection fraction. The left atrium (LA) is anatomically connected with the LV and regulates LV filling through its phasic mechanism that includes reservoir, conduit, and booster function [11–15]. Reservoir strain reflects atrial filling during systole influenced by the descendent of the mitral annulus during systole and LA stiffness. The LA conduit represents the passive LA emptying during the ventricular diastole, and it is dependent on LV stiffness. Finally, the LA booster phase relies on LV late diastolic pressure, and it is closely related to atrial contractility and LA afterload [16, 17]. Different studies reported reservoir and conduit strain impairments in AM patients suggesting diastolic dysfunction during the acute phase of myocarditis [18–22]. In addition, atrial impairment was also related to a worse prognosis in AM patients [21, 23].

Considering the anatomic interaction of LA and LV, we hypothesize that LV fibrosis is associated with LA dysfunction in patients with AM. Therefore, the aim of this study was to analyze the impact of myocardial fibrosis in influencing the LA function in AM patients using CMR.

## Materials and methods

### Study population

In this retrospective, longitudinal, observational, single-center study, all consecutive patients with acute myocarditis who underwent CMR between March 3, 2019, and August 7, 2022, were included. The diagnosis of acute myocarditis was made using the current recommendation of the Position Statement of the European Society of Cardiology Heart Failure Association [2] and for those who met the modified Lake Louise Criteria for the CMR diagnosis of AM according to the Scientific Expert Panel of the American College of Cardiology [6].

Endomyocardial biopsy (EMB) was not performed in our hospital in this low-risk population according to international statement [2]. Low-risk patients were defined by hemodynamic stability, absence of life-threatening arrhythmia, and good response to medical therapy [24, 25].

Exclusion criteria included subjects < 18 years old, previous myocardial infarction, pre-existing cardiomyopathy, and suspected or known prior irreversible myocardial damage. Cardiovascular risk factors were collected from medical records. Hypertension was defined as a systolic blood pressure of ≥ 140 mmHg or a diastolic blood pressure of ≥ 90 mmHg. Smoking status was defined as current smokers and never smokers. Laboratory analyses for cholesterol were conducted according to the standard protocol. The diabetes status was assessed using the World Health Organization criteria [26] or an established diagnosis of type 2 diabetes. Obesity was defined as a BMI > 30 as defined by the World Health Organization criteria [27].

**Fig. 1:**
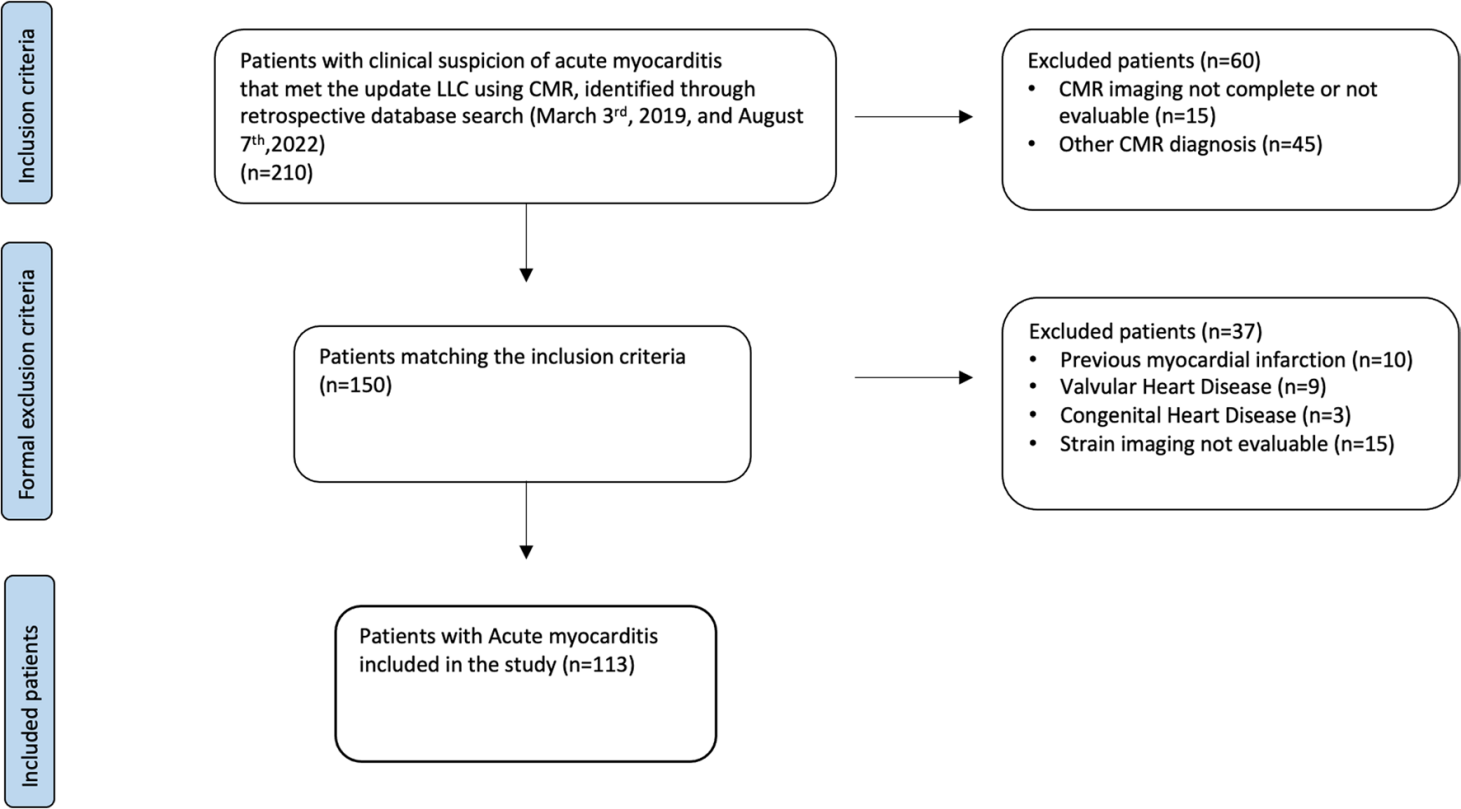
Outline of the study protocol. CMR: cardiovascular magnetic resonance; LLC: Lake Louise criteria

Institutional Review Board approval for this retrospective, cross-sectional study was obtained, and the patients’ consent was waived because of the retrospective nature of the study. A flowchart demonstrating the application of inclusion and exclusion criteria is provided in Fig. 1.

### CMR acquisition

CMR scans were performed at 4.1 ± 2.6 days (median = 1 day, range = 1–10 days) after admission to the hospital by using a Philips Achieva dStream 1.5-T scanner system (Philips Healthcare). Anterior coil arrays were used. All cine images were acquired using a balanced steady-state free precession and retrospective gating during expiratory breath-hold maneuvers (TE: 1.7 ms; TR: 3.4 ms/flip angle: 45°, section thickness = 8 mm) in the long-axis (two-, three-, and four-chamber view) and short-axis plane with whole ventricular coverage from base to apex.

LGE imaging was performed in both long- and short-axis slices 10–12 min after contrast media injection (Gadovist, Bayer Healthcare) with a dose of 0.15 ml per kg body weight using phase-sensitive inversion recovery sequences (PSIR) (TE: 2.0 ms; TR: 3.4 ms; flip angle: 20°, section thickness = 8 mm) with an inversion time determined using the Look-Locker technique. Details of CMR sequence parameters are included in the Supplementary Methods.

### CMR image post-processing

**Fig. 2:**
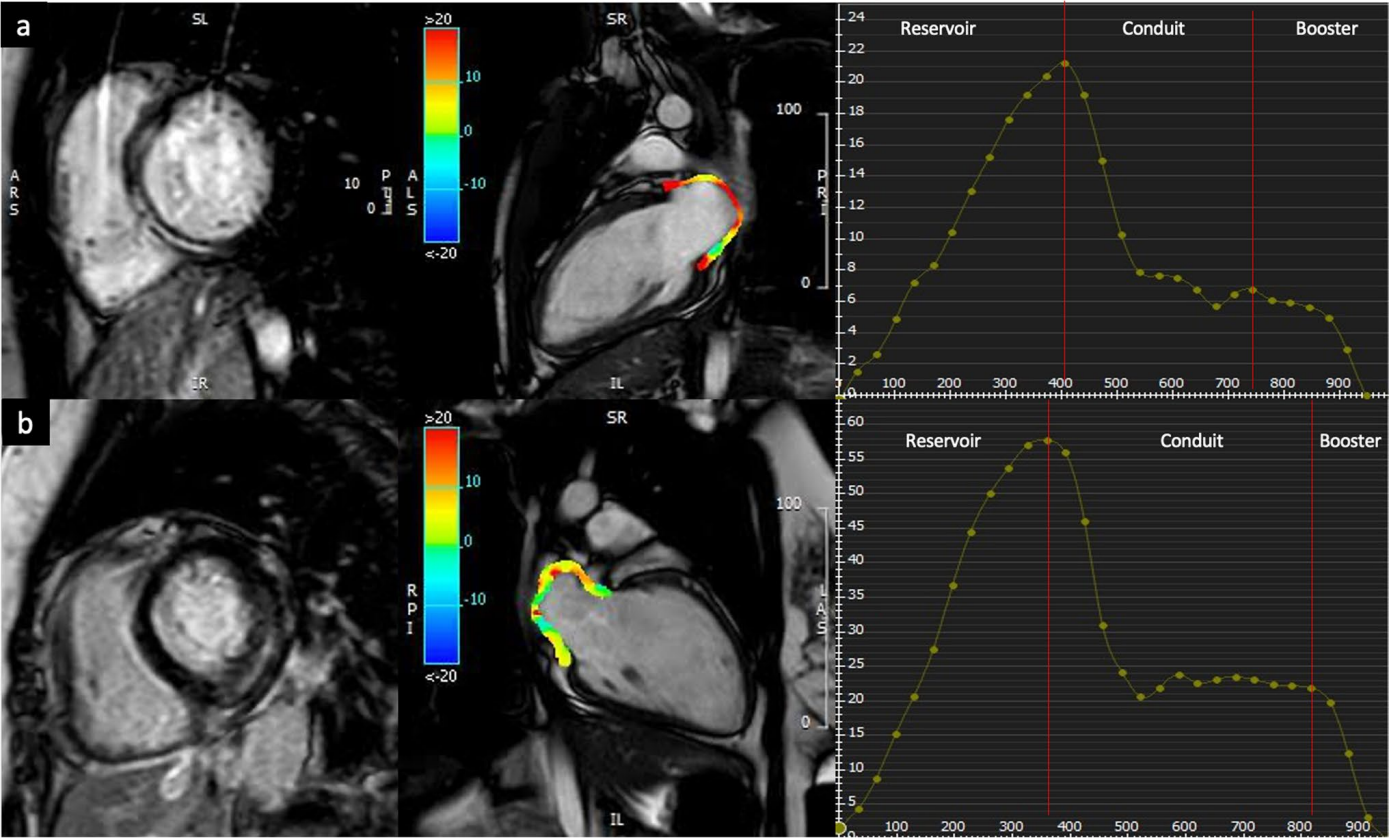
Comparison of LA deformation in an acute myocarditis patient with septal LGE (**a**) and non-septal LGE (**b**). The endo- and epicardial contours of the LA were manually depicted, and the curves of the LA function were automatically obtained. Corresponding LA reservoir, conduit, and booster strain curves in AM patients with septal LGE and infero-lateral LGE. AM: acute myocarditis; CMR: cardiovascular magnetic resonance; LA: left atrium; LGE: late gadolinium enhancement

We used the commercially available software system Circle CVI42 (CVI42, Circle Cardiovascular Imaging Inc.) for CMR tissue tracking (CMR-TT) data analysis. Offline CMR-TT analyses were carried out for the evaluation of atrial deformation. On all the acquired images, LA endocardial borders were manually traced in the long view of the cine images when the atrium was at its minimum volume. In particular, the four-, three-, and two-chamber views were used to derive LA longitudinal strain. After that, with an automatic computation, the software algorithm automatically tracked the myocardial borders throughout the cardiac cycle. The quality of the tracking and contouring was visually validated and manually corrected when needed (Fig. 2).

The biplane area-length was used to determine atrial volume in the LV end systole just before the opening of the mitral valve using the postprocessing software tool Circle CVI42 (CVI42, Circle Cardiovascular Imaging Inc.) [28].

The extent and location of LGE were assessed both qualitatively and quantitatively. Specifically, the evaluation involved counting and determining the location of the affected myocardial segments. The extent and extent of LGE were obtained by tracing the epicardial and endocardial contours in each short-axis image. A region of interest was manually placed in the myocardium without LGE. The LGE was defined as myocardium with mean signal intensity > 5 SDs greater than the reference region of interest [29].

### Statistical analysis

Continuous variables are presented as mean ± standard deviation. Comparisons of continuous data were performed using the independent samples *t* test or Mann–Whitney *U* test; Kolmogorov–Smirnov tests were used to check continuous variables for normal distribution. Categorical variables were compared using the chi-square test or Fisher’s exact test, as appropriate. One-way analysis of variance (ANOVA) or Kruskal–Wallis *H* test with Bonferroni test was used for comparisons of multiple groups, as appropriate. Correlation was assessed using the Pearson *r* and Spearman rho coefficient as appropriate. The association of LA function parameters with myocardial scar extent was assessed using multivariable linear regression analysis. The regression models, with each LA parameter as a dependent variable, included extent of scar as an independent variable and demographic characteristics, traditional cardiovascular risk factors, CMR parameters, and LGE location as covariate.

Similarly, the association of LA function parameters with myocardial scar location was assessed using multivariable linear regression analysis. The regression models, with each LA parameter as a dependent variable, included location of scar as an independent variable and demographic characteristics, traditional cardiovascular risk factors, CMR parameters, and LGE extent as covariate.

To avoid collinearity, correlations between continuous variables were tested using Spearman correlation coefficients and variables with *r* > 0.50 were not included in the same multivariable model.

A *p* value < 0.05 was considered statistically significant. All statistical analysis was performed using IBM SPSS Statistics version 22 (SPSS Inc.).

## Results

### Patient demographics and clinical data

A total of 113 patients with AM, consisting of 89 males (78%) and 24 females (21%) with a mean age of 45.8 ± 17.3 years, were included. By qualitative evaluation, septal LGE was detected in 31 patients with AM (27%). By quantitative evaluation, 67 patients had an amount of LGE ≤ 10%, 33 patients had an amount of LGE between 11 and 19%, and 13 patients showed LGE ≥ 20%. Patients with septal LGE were more often female (*p* = 0.001), older (*p* = 0.002), more frequently hypertensive (*p* = 0.004) and dyslipidemic (*p* = 0.017). Demographic data and clinical characteristics in AM according to the location and extent of LV fibrosis are summarized in Table 1 and Supplementary Table 1.

**Table 1:** Demographic, baseline clinical characteristics, and CMR parameters of the patients enrolled

	All myocarditis (*n* = 113)		
	Septal LGE (*n* = 31)	Infero-lateral LGE (*n* = 82)	*p*
Age, years	56.92 ± 18.89	34.63 ± 15.68	**0.002**
Male, *n* (%)	17 (55%)	72 (87%)	**0.001**
Weight, kg	69.22 ± 14.59	71.65 ± 12.41	0.516
Height, cm	168.40 ± 6.18	172.07 ± 5.57	**0.028**
BSA, m^2^	1.77 ± 0.19	1.83 ± 0.16	0.232
Hypertension, *n* (%)	8 (25%)	12 (15%)	**0.004**
Dyslipidemia, *n* (%)	4 (13%)	8 (10%)	**0.017**
Obesity, *n* (%)	9 (29%)	4 (5%)	0.777
Smoke, *n* (%)	10 (32%)	6 (7%)	0.380
Diabetes, *n* (%)	1 (3%)	4 (5%)	0.070
Familiarity for CAD, *n* (%)	5 (16%)	14 (17%)	0.225
LVEF, %	49.09 ± 12.03	56.18 ± 8.34	**0.009**
LVEDV/BSA, ml/m^2^	97.28 ± 34.05	93.57 ± 21.05	0.522
LVESV/BSA, ml/m^2^	51.69 ± 31.42	43.61 ± 19.06	0.129
LVSV/BSA, ml/m^2^	46.68 ± 9.77	51.14 ± 9.27	0.056
LV mass/BSA, g/m^2^	62.31 ± 17.57	61.10 ± 11.07	0.693
Reservoir, %	25.41 ± 10.99	34.42 ± 11.53	**0.001**
Reservoir rate, s^−1^	1.61 ± 2.40	1.56 ± 0.55	0.871
Conduit, %	12.17 ± 8.02	20.98 ± 9.13	**0.001**
Conduit rate, s^−1^	− 1.28 ± 0.94	− 2.23 ± 0.92	**0.001**
Booster, %	12.89 ± 5	13.39 ± 4.92	0.637
Booster rate, s^−1^	− 1.52 ± 0.62	− 1.52 ± 0.62	0.086
LA volume	11.98 ± 2.16	11.67 ± 2.16	0.719

LA volume was indexed to body surface area. Bold indicates statistical significance

*BSA* body surface area, *CAD* coronary artery disease, *LA* left atrium, *LGE* late gadolinium enhancement, *LVEF* left ventricle ejection fraction, *LVEDV* left ventricle end-diastolic volume, *LVESV* left ventricle end-systolic volume, *LVSV* left volume stroke volume

The LGE pattern along a subepicardial distribution was observed in 48 (42%) AM patients. Mid-wall LGE pattern was observed in 65 (58%) of the enrolled cohort. No patients demonstrated sub-endocardial or transmural distribution. Age increases according to the increase in the extent of LV fibrosis (*p* = 0.045). There were no other significant differences in demographic data and cardiovascular risk factors between the LGE groups.

Patients with septal LGE demonstrated lower LV ejection fraction (*p* = 0.009). There were no other significant differences in CMR parameters between the septal LGE group and non-septal LGE group. LV ejection fraction decreases according to the increase in the extent of LV fibrosis (*p* = 0.012). In addition, LV end-diastolic volume and LV end-systolic volume increase according to the increase in the extent of LV fibrosis (*p* = 0.003 for both). CMR parameters in AM according to the location and extent of LV fibrosis are summarized in Table 1 and Supplementary Table 1.

## LA parameters

LA reservoir strain parameters and conduit functions showed significantly lower values in septal LGE patients as compared with non-septal LGE patients. In contrast, there was no difference in LA reservoir strain rate and contractile booster pump function between the septal LGE group and non-septal LGE group (Table 1). LA strain function, with the exception of the reservoir strain rate, decreased according to the increase in the extent of LV fibrosis (LGE ≤ 10%; LGE 11–19%; LGE ≥ 20%) (Supplementary table 1). The box plots of LA parameters according to the extent of the LV myocardial scar are displayed in Supplementary Fig. 1.

## Association of LA parameters with LV myocardial fibrosis extent

Multivariable linear regression analysis to investigate the association of LV myocardial fibrosis extent with LA strain parameters is summarized in Table 2. For LV myocardial scar extent, the association with reservoir functions and booster functions remained significant: The *β* coefficients were 8.306 (reservoir strain, *p* = 0.007), − 1.002 (reservoir rate, *p* = 0.038), 3.665 (booster strain, *p* = 0.018), and − 0.437 (booster rate, *p* = 0.006) in the demographics and cardiovascular risk factors (model 2; Table 2) adjusted model. In the fully adjusted model, including demographic data, cardiovascular risk factors, CMR parameters, and LV myocardial scar location (model 4; Table 2), there were no associations between LV myocardial scar extent and LA parameters.

**Table 2:** Adjusted associations of LA parameters and extent of myocardial scar

	Model 1		Model 2		Model 3		Model 4	
	*β* coefficient	*p*	*β* coefficient	*p*	*β* coefficient	*p*	*β* coefficient	*p*
Reservoir, %	7.739	**0.028**	8.306	**0.019**	4.672	0.216	2.409	0.239
Reservoir rate, s^−1^	− 1.003	**0.031**	− 1.002	**0.038**	− 1.004	0.510	− 0.943	0.606
Conduit, %	3.130	0.230	3.425	0.197	1.703	0.569	0.855	0.497
Conduit rate, s^−1^	− 0.414	0.107	− 0.455	0.093	− 0.315	0.691	− 0.229	0.519
Booster, %	3.613	**0.022**	3.665	**0.018**	3.135	0.061	2.375	0.168
Booster rate, s^−1^	− 0.524	**0.007**	− 0.437	**0.006**	− 0.374	0.063	− 0.219	0.262

The *β* coefficient expresses the mean difference in each LA parameter and extent of LV LGE, after adjustment for the covariates in each model. Model 1 was adjusted for demographic data. Model 2 was adjusted for demographic data and cardiovascular risk factors. Model 3 was adjusted for demographic data, cardiovascular risk factors, and CMR parameters. Model 4 was adjusted for demographic data, cardiovascular risk factors, CMR parameters, and LV LGE location. Demographic data included age and sex. Cardiovascular risk factors included body mass index, hypertension, dyslipidemia, diabetes, familiarity for coronary artery disease, and smoking status. CMR parameters included LVEF and LV volumes. Bold indicates statistical significance

## Association of LA parameters with LV myocardial fibrosis location

Multivariable linear regression analysis to investigate the association of LV myocardial fibrosis location with LA strain parameters is summarized in Table 3. For LV myocardial scar location, the associations with reservoir and conduit function remained significant: The *β* coefficients were 7.116 (reservoir strain, *p* = 0.024) and 5.185 (conduit strain, *p* = 0.026) in the fully adjusted model (model 4; Table 3).

**Table 3:** The *β* coefficient expresses the mean difference in each LA parameter and location of myocardial fibrosis, after adjustment for the covariates in each model

	Model 1		Model 2		Model 3		Model 4	
	*β* coefficient	*p*	*β* coefficient	*p*	*β* coefficient	*p*	*β* coefficient	*p*
Reservoir, %	7.919	**0.003**	9.747	**0.001**	8.024	**0.008**	7.116	**0.024**
Reservoir rate, s^−1^	− 0.514	0.118	− 0.523	0.154	− 0.282	0.076	− 0.277	0.381
Conduit, %	5.479	**0.009**	6.609	**0.002**	5.436	**0**.**025**	5.185	**0.026**
Conduit rate, s^−1^	− 0.502	**0.011**	− 0.578	**0.006**	− 0.374	0.097	− 0.332	0.179
Booster, %	1.545	0.219	2.574	**0.041**	2.637	0.053	2.340	0.096
Booster rate, s^−1^	− 0.307	**0.047**	− 0.390	**0.014**	− 0.390	**0.018**	− 0.351	0.105

Model 1 was adjusted for demographic data. Model 2 was adjusted for demographic data and cardiovascular risk factors. Model 3 was adjusted for demographic data, cardiovascular risk factors, and CMR parameters. Model 4 was adjusted for demographic data, cardiovascular risk factors, CMR parameters, and LV LGE extent. Demographic data included age and sex. Cardiovascular risk factors included body mass index, hypertension, dyslipidemia, diabetes, familiarity for coronary artery disease, and smoking status. CMR parameters included LVEF and LV volumes. Bold indicates statistical significance

## Discussion

As far as we are aware, there has been no previously established evidence in the literature regarding the correlation between LA strain functions and LV myocardial fibrosis in AM patients. The main results of the current study can be summarized as follows: (1) the presence of a myocardial scar, detected by LGE, influences the LA mechanism; (2) in the fully adjusted model, LA dysfunction is related to LV LGE location, but not to LV LGE extent; and (3) septal LGE was associated with lower reservoir and conduit strain.

The relationship between the myocardial scar and atrial function has been analyzed in the Multi-Ethnic Study of Atherosclerosis showing that the presence of LV LGE leads to a reduced LA mechanism [30]. Overall, our results suggested that it is the location of LGE itself more than the extent of the myocardial scar that is the main determinant of atrial impairment. Indeed, differences in the myocardial fibrosis pattern may have a different impact on heart chamber function and mechanism [31]. Septal LGE determines a predominant dysfunction in subepicardial and transmural myocardial fibers regardless of the extent of myocardial fibrosis, suggesting an impairment in myocardial stiffness and LV filling [31, 32]. Due to the anatomical communication of the cardiac chambers, LA strain is heavily influenced by the LV mechanism [16].

Studies comparing left atrial strain in patients with myocarditis have demonstrated impaired passive atrial function with a preserved booster pump mechanism in AM patients [18, 19]. However, little is known about the relationship between the LA mechanism and myocardial fibrosis. Given the association between myocardial fibrosis and adverse outcome in AM, the identification of factors related to myocardial fibrosis may help to risk stratify patients. It is possible to hypothesize that septal LGE occurring in myocarditis can result in structural changes in the LV that may affect atrial compliance, which is essential for proper LA reservoir and conduit strain function. Conversely, LA booster pump function may act as a compensatory mechanism to maintain LV filling [11, 20, 33]. These results are consistent with a transgenic mouse model that demonstrated altered cardiac performance and reduced cardiac elasticity both in vivo and in vitro [34]. Conversely, we did not find a significant association between atrial function and the extent of myocardial fibrosis in multivariable linear regression analysis. This may be explained by the predominant role of LGE location compared to its extent, potentially involving the cardiac conduction system and resulting in atrioventricular conduction delay.

To the best of our knowledge, this is the first work focused on the impact of myocardial scar on atrial function in AM patients using CMR. Our data support the hypothesis that myocardial damage in the acute phase of myocarditis leads to atrial dysfunction. Confirming these results in a larger cohort of patients may contribute to a more in-depth understanding of myocarditis pathophysiology, which would ideally lead to an earlier therapeutic approach and a better outcome for these patients.

In this study, there are some limitations: first, the sample size was relatively small, and the study was retrospective in nature. Although our study yielded promising results, it is essential to conduct further prospective trials involving a larger patient cohort to validate our findings. The second limitation of this study is that there was no systematic endomyocardial biopsy to detect acute myocarditis. Third, we did not evaluate the predictive value of strain for adverse cardiovascular events at follow-up. Furthermore, the amount of LGE can change throughout the natural course of myocarditis, potentially leading to an overestimation due to concomitant myocardial edema in the acute phase. Future prospective studies are needed to assess the relationship between atrial strain and myocardial fibrosis at different stages of the natural course of myocarditis.

## Conclusion

In patients with acute myocarditis, left atrial function abnormalities are significantly associated with LV LGE location. Septal LGE is paralleled by a deterioration of LA reservoir and conduit function. Such findings may provide new insight into the pathophysiology of acute myocarditis by suggesting the impact of LGE location in atrial dysfunction. Further longitudinal studies are warranted to confirm these results.

### Supplementary information

Below is the link to the electronic supplementary material.

Supplementary file1 (PDF 220 KB)

## Abbreviations

AM: Acute myocarditis
CMR: Cardiovascular magnetic resonance
CMR-TT: CMR tissue tracking
EMB: Endomyocardial biopsy
LA: Left atrium
LGE: Late gadolinium enhancement
LV: Left ventricle

## Declarations

### Guarantor

The scientific guarantor of this publication is Luca Saba.

### Statistics and biometry

No complex statistical methods were necessary for this paper.

### Study subjects or cohorts overlap

The authors state that none of its contents have been published previously.

### Methodology

• retrospective

• observational

• performed at one institution
