# Supplementary material for: Effect of late gadolinium enhancement on left atrial impairment in myocarditis patients
Source: Eur Radiol. 2023 Sep 2;34(3):1846–53. doi: 10.1007/s00330-023-10176-3 (PMC10873434; doi:10.1007/s00330-023-10176-3)
Supplement: Supplementary file 1 — Supplementary file1 (PDF 220 KB) [file 330_2023_10176_MOESM1_ESM.pdf]

Effect of Late Gadolinium Enhancement on

Left Atrial Impairment in Myocarditis Patients

Electronic Supplementary Material (ESM)

Eur Radiol (2023) Cau R, Muscogiuri G, Pisu F et al.

Sequence parameters

All cine-images were acquired using a balanced steady-state free precession and retrospective gating during an expiratory breath- hold manoeuvres (TE: 1.7mssec; TR: 3.4msec; flip-angle: 45°, section thickness = 8 mm) in long-axis (two-, three- and four-chamber view) and short-axis plane with whole ventricular coverage from base to apex.

T2-STIR images were obtained using triple inversion recovery T2-weighted pulse sequence (TR = 2 RR, TE ≈ 70 msec; flip-angle: 45°, section thickness = 8 mm, FOV 300×300 mm2) in long-axis (two-, three- and four-chamber view) and short-axis plane with whole ventricular coverage from base to apex

T1 mapping was performed in the short-axis plane in three slices (at the base, mid-ventricular, and apex, respectively) using a single-breath-hold, ECG-triggered, MOLLI sequence before contrast media injection (TE 1.1 msec; TR 2.5 msec; flip angle 35°; FOV, 300 × 300 mm2).

T2 mapping was acquired before the administration of contrast-media on three representative short-axis slices (at the base, mid-ventricular, and apex, respectively) using a single-breath-hold, black-blood prepared ECG-triggered, spin-echo multiecho sequence.

LGE imaging was performed in both long and short axis slices 10-12 minutes minutes after contrast media injection (Gadovist, Bayer Healthcare, Berlin, Germany) with a dose of 0.15 ml per kg body weight using phase-sensitive inversion recovery sequences (PSIR) (TE: 2.0 msec; TR: 3.4 msec; flip-angle: 20°, section thickness = 8 mm) with an inversion time determined using the Look- Locker technique.

Eur Radiol (2023) Cau R, Muscogiuri G, Pisu F et al.

Supplemental Table 1

	All myocarditis (n=113)			
	Mild LGE (n= 67)	Moderate LGE (n= 33)	Severe LGE (n= 13)	p
Age, y	35,79 ± 17,06	44,77 ± 20,44	47,36 ± 24,93	**0,045**
Male, n (%)	50 (75%)	21 (63%)	11	0,227
Weight, kg	70,07 ± 13,90	69,31 ± 13,93	73,87 ± 9,12	0,718
Height, cm	170,78 ± 6,60	170,40 ± 6,26	171 ± 6,14	0,642
BSA, m^2	1,80 ± 0,19	1,78 ± 0,18	1,87 ± 0,10	0,564
Hypertension, n (%)	8 (12%)	6 (18%)	2 (15%)	0,521
Dyslipidemia, n (%)	8 (12%)	8 (24%)	8 (61%)	0,622
Obesity, n (%)	7 (10%)	4 (12%)	1 (8%)	0,846
Smoke, n (%)	7 (10%)	6 (18%)	1 (8%)	0,342
Diabetes, n (%)	2 (3%)	1 (3%)	1 (8%)	0,655
Familiarity for CAD, n (%)	10 (15%)	10 (30%)	3 (23%)	0,085
LVEF, %	55,37 ± 8,66	54,78 ± 10,19	45,44 ± 12,09	**0,012**
LVEDV/BSA, ml/m^2	90,10 ± 22,37	96,07 ± 20,65	120,41 ± 40,12	**0,003**
LVESV/BSA, ml/m^2	43,10 ± 20,18	44,93 ± 19,56	67,60 ± 40,22	**0,003**
LVSV/BSA, ml/m^2	50,60 ± 9,74	51,11 ± 8,22	52,78 ± 14,35	0,827
LV mass/BSA, g/m^2	59,58 ± 11,35	65,05 ± 12,11	64,36 ± 14,13	0,14

Reservoir, %	34,97 ± 12,32	30,37 ± 9,92	22,85 ± 9,20	**0,004**
Reservoir rate, s−1	1,58 ± 0,58	1,39 ± 0,48	2,22 ± 3,93	0,232
Conduit, %	21,71 ± 10,10	16,57 ± 7,35	13,82 ± 7,83	**0,017**
Conduit rate, s−1	-2,29 ± 1,03	-1,73 ± 0,89	-1,36 ± 0,92	**0,004**
Booster, %	13,77 ± 4,87	13,41 ± 4,31	9,26 ± 4,01	**0,014**
Booster rate, s−1	-1,80 ± 0,59	-1,66 ± 0,48	-1,18 ± 0,51	**0,004**
LA volume	11,45 ± 2,90	12,81 ± 1,89	11,98 ± 2,34	0,606

Supplemental Table 1: Demographic. baseline clinical characteristics, and CMR findings according to the extent of myocardial fibrosis.

The p value shows the ANOVA value between the three groups.

BSA: body surface area; CAD: coronary artery disease; LA: Left Atrium; LGE: Late gadolinium enhancement; LVEF: Left Ventricle Ejection Fraction; LVEDV: Left Ventricle End-Diastolic Volume; LVESV: Left Ventricle End-Systolic Volume; LVSV: Left Volume Stroke Volume.

LA volume was indexed to body surface area.

Bold indicates statistical significance.

Supplemental Figure 1

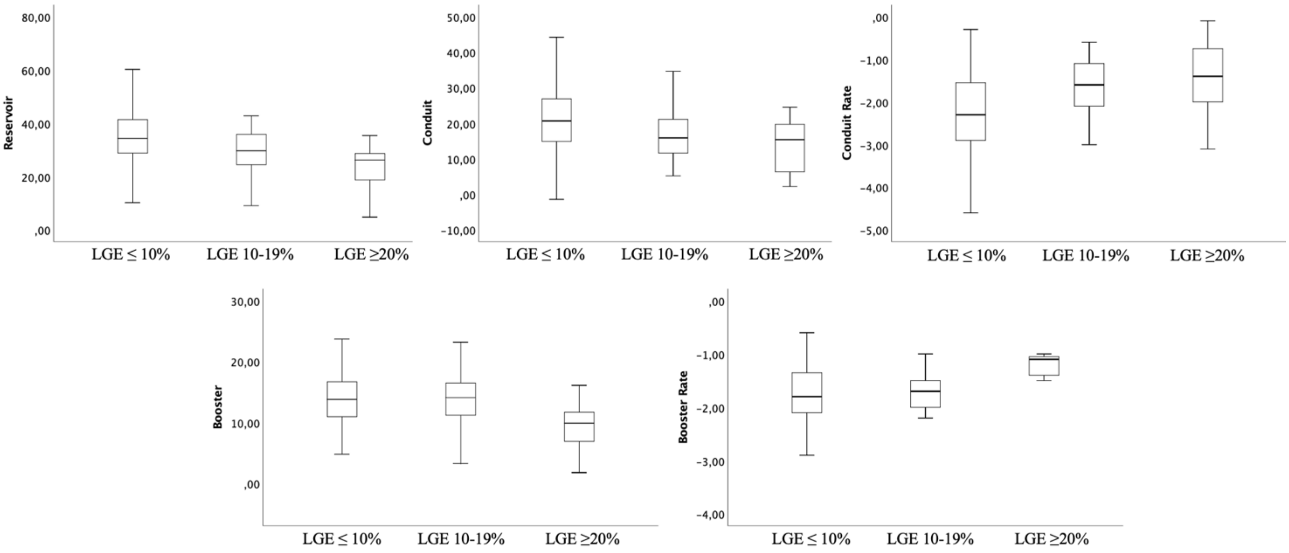

Supplementary Figure 1: LA dynamics in acute myocarditis patients. Box plots comparing LA functions between acute myocarditis patients according to the extent of LV enhancement.
